# Supplementary material for: Modeling glioblastoma heterogeneity as a dynamic network of cell states
Source: Mol Syst Biol. 2021 Sep 16;17(9):e10105. doi: 10.15252/msb.202010105 (PMC8444284; doi:10.15252/msb.202010105)
Supplement: Supplementary file 6 — Source Data for Figure 5 [file MSB-17-e10105-s004.zip › Figure5A_sourcedata/GSEA_3071/hallmarks_stateVI.GseaPreranked.1623742557031/HALLMARK_INFLAMMATORY_RESPONSE.html]

Details for gene set HALLMARK\_INFLAMMATORY\_RESPONSE[GSEA]

|  || Dataset | state63071 |
| Phenotype | NoPhenotypeAvailable |
| Upregulated in class | na\_pos |
| GeneSet | HALLMARK\_INFLAMMATORY\_RESPONSE |
| Enrichment Score (ES) | 0.3715293 |
| Normalized Enrichment Score (NES) | 2.177657 |
| Nominal p-value | 0.0 |
| FDR q-value | 0.002166234 |
| FWER p-Value | 0.005 |
Table: GSEA Results Summary

  

Fig 1: Enrichment plot: HALLMARK\_INFLAMMATORY\_RESPONSE      
 Profile of the Running ES Score & Positions of GeneSet Members on the Rank Ordered List

  

| PROBE | GENE SYMBOL | GENE\_TITLE | RANK IN GENE LIST | RANK METRIC SCORE | RUNNING ES | CORE ENRICHMENT || 1 | SERPINE1 |  |  | 0 | 1.673 | 0.0601 | Yes |
| 2 | DCBLD2 |  |  | 16 | 1.066 | 0.0943 | Yes |
| 3 | F3 |  |  | 26 | 0.988 | 0.1273 | Yes |
| 4 | AXL |  |  | 33 | 0.946 | 0.1596 | Yes |
| 5 | PLAUR |  |  | 34 | 0.936 | 0.1933 | Yes |
| 6 | EDN1 |  |  | 50 | 0.866 | 0.2202 | Yes |
| 7 | MYC |  |  | 69 | 0.815 | 0.2445 | Yes |
| 8 | INHBA |  |  | 80 | 0.781 | 0.2698 | Yes |
| 9 | SPHK1 |  |  | 141 | 0.677 | 0.2774 | Yes |
| 10 | HBEGF |  |  | 172 | 0.627 | 0.2916 | Yes |
| 11 | MET |  |  | 174 | 0.626 | 0.3138 | Yes |
| 12 | ADORA2B |  |  | 192 | 0.608 | 0.3309 | Yes |
| 13 | IL18 |  |  | 239 | 0.572 | 0.3386 | Yes |
| 14 | ADRM1 |  |  | 256 | 0.561 | 0.3544 | Yes |
| 15 | RIPK2 |  |  | 267 | 0.555 | 0.3715 | Yes |
| 16 | CD55 |  |  | 452 | 0.471 | 0.3372 | No |
| 17 | LPAR1 |  |  | 575 | 0.432 | 0.3188 | No |
| 18 | TNFSF10 |  |  | 631 | 0.416 | 0.3184 | No |
| 19 | IL7R |  |  | 718 | 0.392 | 0.3086 | No |
| 20 | IRF1 |  |  | 771 | 0.382 | 0.3079 | No |
| 21 | PVR |  |  | 839 | 0.370 | 0.3025 | No |
| 22 | KLF6 |  |  | 854 | 0.368 | 0.3118 | No |
| 23 | ADM |  |  | 1185 | 0.318 | 0.2314 | No |
| 24 | EMP3 |  |  | 1278 | 0.306 | 0.2168 | No |
| 25 | SLC7A2 |  |  | 1347 | 0.298 | 0.2086 | No |
| 26 | IL15 |  |  | 1353 | 0.297 | 0.2179 | No |
| 27 | RHOG |  |  | 1355 | 0.297 | 0.2283 | No |
| 28 | HIF1A |  |  | 1458 | 0.284 | 0.2101 | No |
| 29 | CD70 |  |  | 1489 | 0.281 | 0.2119 | No |
| 30 | RELA |  |  | 1791 | 0.252 | 0.1371 | No |
| 31 | LIF |  |  | 1804 | 0.250 | 0.1428 | No |
| 32 | TAPBP |  |  | 1817 | -0.252 | 0.1485 | No |
| 33 | IFNAR1 |  |  | 1897 | -0.263 | 0.1360 | No |
| 34 | HAS2 |  |  | 2025 | -0.282 | 0.1108 | No |
| 35 | MMP14 |  |  | 2440 | -0.365 | 0.0086 | No |
| 36 | KIF1B |  |  | 2443 | -0.365 | 0.0212 | No |
| 37 | CALCRL |  |  | 2853 | -0.478 | -0.0754 | No |
| 38 | PDE4B |  |  | 2948 | -0.513 | -0.0832 | No |
| 39 | TNFAIP6 |  |  | 3085 | -0.578 | -0.1002 | No |
| 40 | ITGB8 |  |  | 3148 | -0.616 | -0.0953 | No |
| 41 | SRI |  |  | 3179 | -0.643 | -0.0806 | No |
| 42 | RGS16 |  |  | 3304 | -0.765 | -0.0876 | No |
| 43 | BTG2 |  |  | 3377 | -0.857 | -0.0768 | No |
| 44 | CD82 |  |  | 3408 | -0.912 | -0.0524 | No |
| 45 | PDPN |  |  | 3580 | -1.590 | -0.0429 | No |
| 46 | SLC1A2 |  |  | 3589 | -1.634 | 0.0136 | No |
Table: GSEA details [plain text format]

  

Fig 2: HALLMARK\_INFLAMMATORY\_RESPONSE: Random ES distribution      
 Gene set null distribution of ES for **HALLMARK\_INFLAMMATORY\_RESPONSE**
